# Evaluation of sensitivity and specificity of ELISA against Widal test for typhoid diagnosis in endemic population of Kathmandu

**DOI:** 10.1186/s12879-015-1248-6

**Published:** 2015-11-14

**Authors:** Anurag Adhikari, Ramanuj Rauniyar, Pramod Prasad Raut, Krishna Das Manandhar, Birendra Prasad Gupta

**Affiliations:** Asian Institute of Technology & Management, Lalitpur, Nepal; Capital Hospital and Research Center, Kathmandu, Nepal; Central Department of Biotechnology, Tribhuvan University, Kirtipur, Kathmandu, Nepal; Everest Institute of Virology and Immunology, Kathmandu, Nepal

**Keywords:** Typhoid Fever, *Salmonella typhi*, Widal test, ELISA, Sensitivity, Specificity, Nepal

## Abstract

**Background:**

Widal test, which has poor predictive outcomes in predominant typhoid population, is not standard enough to predict accurate diagnosis. This study aims to compare the diagnostic accuracy of Widal test to ELISA using blood culture as gold standard.

**Methods:**

The blood samples were collected in Capital Hospital, Kathmandu, Nepal from febrile patients having ≥48 h fever in 3 years study period for blood culture, Widal test and IgG-IgM ELISA.

**Results:**

Amongst 1371 febrile cases, 237 were *Salmonella typhi* positive to blood culture and 71.4 % typhoid fever patient were of 46–60 years old with male to female ratio of 2:1. Blood culture confirmed patients had ≥1:40 anti-TH and anti-TO titre in 45.56 % (*n* = 108) and 43.88 % (*n* = 104) patients respectively. The sensitivity and specificity of IgG (0.96 and 0.95) and IgM (0.95 and 0.94) at 95 % confidence level were significant compared to Widal anti-TH (0.72 and 0.58) and TO (0.80 and 0.51) test (p value, 0.038) at titre level ≥1:200. Further the PPV of Widal TH and TO (0.38 and 0.23) was low compared to IgG and IgM ELISA (0.78 and 0.77) (p value, 0.045).

**Conclusion:**

Widal test is not sensitive enough for an endemic setting like Nepal and thus should be either replaced with more accurate test like ELISA or follow an alternative diagnostic methodology.

## Background

Typhoid fever is becoming a key health problem in developing countries. Lack of safe and clean drinking water supplies to the people and adequate sewage disposal are the major reasons [1]. The fever has been one of the leading diagnosed fever ailment among the fever related caseses in most of the hospitals of Nepal [2]. It is popularly known as ‘Bisham Joro’ in local language meaning the ‘fever with poison’. Typhoid is prevalent in mid-hills, valleys and southern belts as an endemic disease with peak incidence in between April and August [3, 4]. Outbreaks are more common in summer season affecting mainly the children in Kathmandu [5–7]. Diagnosis is done by culture and immunological tests, however, isolation of the etiological agent, the *Salmonella enterica* (serovar typhi) from bone marrow culture is an ideal gold standard [8]. Invasive procedure and unavailability of culture facilities in rural health centers are chief limiting factors. Besides, the widely used blood culture has poor sensitivity of 40–60 % making room for false negative results to around half of the typhoid patients [9]. In addition, the requirement of 3 days time duration for diagnosis delays the line of treatment loosing applicability of early diagnosis. Further, usage of the antibiotics prior to hospital admittance, which is a common practice in Nepal, cause poor sensitivity to the culture base diagnosis [10]. In spite of the all, blood culture technique is still the gold standard in the febrile cases of typhoid. Routine Widal test is alternatively adopted second most popular choice for diagnosis as blood culture remains controversial due to its biased diagnosis [11]. Enzyme-linked immunosorbent assay (ELISA) based diagnosis has also been studied previously with good diagnostic accuracy [12–15]. This study aims to compare diagnostic accuracy of Widal test and ELISA in febrile patients taking blood culture as gold standard tool for the diagnosis of typhoid fever.

## Material and methods

### Patients, inclusion/exclusion criteria and data analysis

**Fig. 1:**
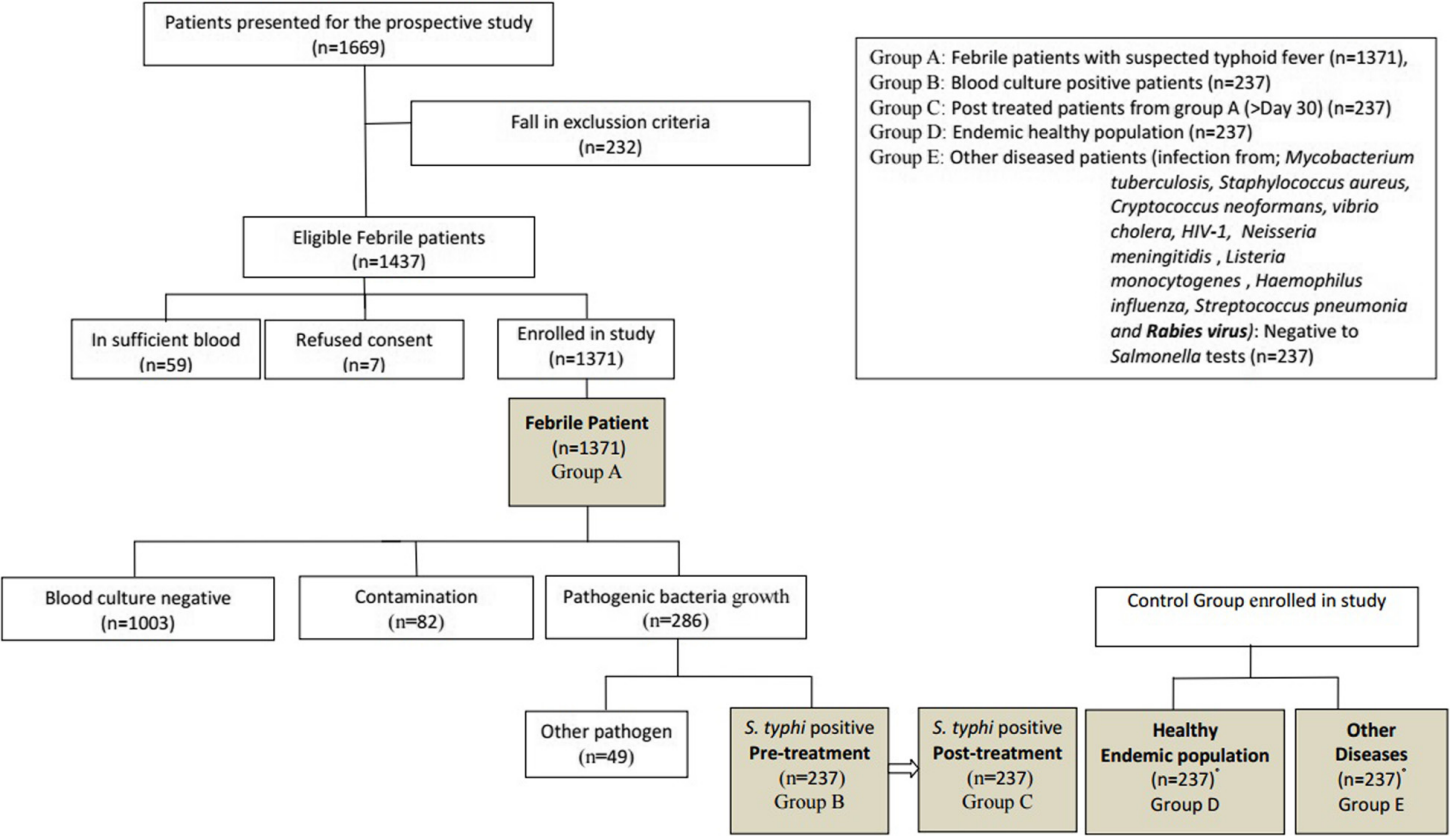
Consort chart for patients in study. Group A: Febrile patients with suspected typhoid fever (*n* = 1371), Group B: Blood culture positive patients (*n* = 237), Group C: Post treated patients from group A (>Day 30) (*n* = 237), Group D: Endemic healthy population (*n* = 237), Group E: Other diseased patients (infection from; *Mycobacterium tuberculosis, Staphylococcus aureus, Cryptococcus neoformans, Vibrio cholera, HIV-1, Neisseria meningitidis*, *Listeria monocytogenes*, *Haemophilus influenza, Streptococcus pneumonia and Rabies virus)*: Negative to *Salmonella* tests (*n* = 237)

Patients attending Capital Hospital, a centrally located hospital at Kathmandu, during the months January 2011 to December 2013 with complaint of fever over 72 h without obvious focus of infection and clinical suspicion of typhoid fever (high fever, malaise, headache, constipation or diarrhoea) were prospectively enrolled in this study. Patients were divided into five age groups i.e. 1–15 (children), 16–30 (young), 31–45 (young adults), 46–60 (adults) and >60 years (olds). Pregnant women were excluded from the study. The ELISA and Widal tests were performed by a single specialized clinical researcher who was blind to the patient's diagnosis in reference to standard throughout the study period. The patients with febrile cases were screened when found positive to reference standard (Blood Culture). The control populations with negative blood culture reports were also included in the study, so as to make self evaluation of reference standard. The sample population inclusive of blood culture positive was grouped into five different groups (Fig. 1).

### Blood collection and bacterial culture for pathogen isolation

Blood were collected in BACTEC Peds Plus™**/**F culture vials and immediately (within 10 min) transferred to laboratory to be loaded in Bactec 9240 (Becton Dickinson, USA) culture instrument for growth. Blood isolates which were found culture positive were reconfirmed for *Salmonella* by slide agglutination, using monospecific anti sera (Sifin, Germany) as described by the Kauffmann-White scheme [16–18].

### Quantitative Widal test

The Widal tube agglutination test was performed using Wellcolex®(Remel, UK) in the group A (*n* = 1371) as well as for group B, C D and E according to the manufacturer's instructions parallel to the blood culture procedure of individual groups. Briefly, serum remained after keeping for blood culture was diluted in 0.86 % saline solution starting with 1:100. *Salmonella* ‘O’ and ‘H’ antigens from the standard preparation were added and the tubes were incubated at 37 °C for 1 h. After incubation, the tubes were centrifuged for 5 min, and respective agglutinations were observed. The Widal TO/TH titre were taken as the highest dilution of serum with visible agglutination. The cut off titre set for *Salmonella typhi* anti O was >1:80 while it was >1:160 for anti H in this study [11].

### Enzyme Linked Immunosorbent Assay (ELISA)

Similar to the Widal test, Anti-IgM and anti-IgG sandwich ELISA (MyBioSource, Inc. CA, USA) was done in all the groups. Briefly, 100 μl of coating antigen (1 μg/ml) diluted in antigen coating buffer (Immunochemistry, MN, USA) were dispensed in Nunc-Immuno 96 MicroWell solid plates (Thermo Fisher Scientific, USA) along with negative control (Only coating buffer) according to plan. The plates were incubated at 4 °C overnight and the wells were blocked using 1 % bovine serum albumin (BSA) prepared in phosphate buffer saline (PBS). The plates were washed by 125 μl washing buffer (0.1%BSA with Tween20). Hundred microliter of serially diluted sera (1:200 to 1:3200) in PBS-BSA was dispensed to each well and incubated at 25 °C for 4 h. After washing, 100 μl detector antibody conjugated with horse reddish peroxidase (HRP) was added in dilutions (1:500 anti IgA, 1:5000 anti IgG, 1:2500 anti IgM) and incubated for 30 min at 25 °C. After that, 100 μl trimethyle benzidine (TMB) substrate was added and incubated for 15 min at dark. The reaction was stopped by addition of 1 N H_2_SO_4_ to measure optical density (OD) at 450 nm in ELISA plate reader (Bio-Rad). Cut off values were assessed following the mean ± SD of the OD from healthy endemic controls of group D which was 0.3 for IgG and 0.2 for IgM.

### Statistical analysis

The positive predictive value is the principle finding of this study which elucidates the efficacy of current diagnostic assays. The sensitivity, specificity and predictive values were calculated as described earlier [19]. Receiver operating Characteristics (ROC) used to establish cut off titre, was determined by plotting specificity (x-axis) versus sensitivity (y-axis) at all tested cut off titres. The highest number of true positive and the lowest false positive results were used to represent cut off values by comparing the points with maximum Youden index (*J* = max_*c*_ [Se (*c*) + Sp (*c*) – 1]). Collected data were analyzed and interpreted statistically using graphPad prism version 6.0 and SPSS 17.0. Normal distribution of data sets were analysed by Kolmogorov-Smirnov test. All the values are expressed as mean ± SD and are analyzed using Student’s *t* test which is parametric as well Mann–Whitney test wherever applicable. A value (p value, <0.05) was considered significant unless stated otherwise. Sensitivity, specificity and predictive values were calculated as described elsewhere [20, 21]

### Ethical statement

The study was approved by the Capital Hospital Ethical Review Board (CHRB). The adult participants gave written informed consent while children below 18 year were enrolled only after the written informed consent from their guardian was received.

## Results

### Demographic and geographical distribution

The 1371 patients, enrolled in the study held between January 2011 and December 2013, had representation from all the three geographical regions of Nepal *viz*. Mountain, Hill and Terai, as Kathmandu, the capital city, hosts a broad range of people from all over the country. The prevalence of typhoid cases in Terai region was highest (69 %; p value, 0.03) in comparison to mid hill regions around Kathmandu valley (30 %) and the least in mountain region (1.39 %) (Table 1). There were 400 females and 971 males who consented for this study. The infection was found more in males of 16–30 years (median age, 21.5 years) age group with male–female ratio of 2:1, among the positive cases (*n* = 237) of typhoid as confirmed by blood culture, and was followed by age groups of 1–15, 31–45, 46–60 and >60 year.

**Table 1:** Distribution of Typhoid patients according to regions and sex

Geographical Regions	Patients		
	Male	Female	Total
Terai Region (<2297 ft)	621 (65.92 %)	321 (34.07 %)	942 (68.71 %)*
Hill Region (2000–10000 ft)	340 (82.92 %)	70 (17.07 %)	410 (29.91 %)
Mountain Region (>10000 ft)	10 (52.63 %)	9 (47.36 %)	19 (1.39 %)

*p value, <0.05 while comparing the patients from Hill region

### Pathogen distribution among febrile cases

Out of 1371 cases, only 237 samples (17.28 %) showed positive growth of *S. typhi* in the blood culture and 120 patients were admitted in the hospital for treatment. The cultures also had co-infection of *Actinobacteria (n = 3, 2.5 %)* and *Klebsiella pneumonia* (*n* = 2, 1.67 %) in the admitted inpatients of the typhoid fever (Table 2). In 237 patients, *Actinobacteria* (0.21 %) was found only in male while *Klebsiella pneumoniae* (0.14 %) were found only in female. Pathogen other than *Salmonella* was isolated from 49 patients (Fig. 1).

**Table 2:** Culture report of *S.typhi* positive cases and co-infection with other pathogens among inpatient and outpatient of capital hospital

	Inpatients	Outpatients
*Salmonella typhi*	115 (95.83 %)	122 (100 %)
*Actinobacteria*	3 (2.5 %)^*^	0 (0 %)
*Klebsiella pneumonia*	2 (1.67 %)^*^	0 (0 %)

^*^Co-infection with *S.typhi*

### Clinical symptoms of the disease

Different symptoms were found in the typhoid positive cases. The absolute symptom was fever (100 %; p value, 0.04) however, other major symptoms observed were headache (97.47 %; p value, <0.034), loss of appetite (90.3 %; p value, 0.028) and chill (74.26 %; p value, 0.034). Abdominal discomfort, myalgia, vomiting, constipation, rigor, diarrhea, and dysuria were other symptoms observed in less than half of culture confirmed typhoid patient. Hence, fever, headache and loss of appetite were significant symptoms for the fever ailment (Table 3).

**Table 3:** Clinical sign and symptoms distribution

S.N.	Symptom expressed by patient	*Salmonella typhi (n)*	Percentage
1	Fever	237	100.00
2	Headache	231	97.47
3	Loss of appetite	214	90.30
4	Chills	176	74.26
5	Abdominal Discomfort	109	45.99
6	Myalgia	99	41.77
7	Vomiting	89	37.55
8	Constipation	64	27.00
9	Rigor	55	23.21
10	Diarrhea	45	18.99
11	Dysuria	42	17.72

### Qualitative slide agglutination Widal test

Widal test used as the primary screening assay by typing O and H antigen of *Salmonella* showed overall positivity rate of 21.74 % and 22.68 % respectively at the titre ≥1:40. (Table 4). Among culture confirmed patients, group A (*n* = 237), the number of TH (*n* = 108) and TO (*n* = 104) positive at titre ≥1:40 was significantly lower (p value, 0.04) than from the culture negative patients (*n* = 1134) group. Though blood culture showed negative to typhoid test, Widal test was found positive to TH antigen (*n* = 203, 17.92 %) and TO antigen (*n* = 194, 17.11 %) test at same titre (Table 4).

**Table 4:** Number of anti TH and anti TO levels in blood isloates

Titration Record	Total Patients (*n* = 1371)	Culture positive patients (*n* = 237)	Culture negative patients (*n* = 1134)
Anti TH			
Aggutinition	311 (22.68 %)	108 (45.57 %)	203 (17.92 %)
≥1:640	78 (25.08 %)	36 (33.33 %)	42 (20.69 %)
1:320	156 (50.16 %)	55 (50.93 %)	101 (49.75 %)
1:160	176 (56.59 %)	89 (82.41 %)	87 (42.86 %)
1:80	201 (64.63 %)	98 (90.74 %)	103 (50.74 %)
1:40	311 (100 %)	108 (100 %)	203 (100 %)
No Agglutination	1060 (77.32 %)	129 (54.43)	931 (82.14 %)
Anti TO			
Aggutinition	298 (21.74 %)	104 (43.88 %)	194 (17.11 %)
≥1:640	71 (23.83 %)	29 (27.88 %)	42 (21.65 %)
1:320	147 (49.33 %)	45 (43.27 %)	102 (52.58 %)
1:160	164 (55.03 %)	84 (80.77 %)	80 (41.24 %)
1:80	198 (66.44 %)	91 (87.5 %)	107 (55.15 %)
1:40	298 (100 %)	104 (100 %)	194 (100 %)
No Agglutination	1073 (78.26 %)	133 (56.12 %)	940 (82.89 %)

### Assessment of ELISA for the diagnosis of typhoid fever

**Fig. 2:**
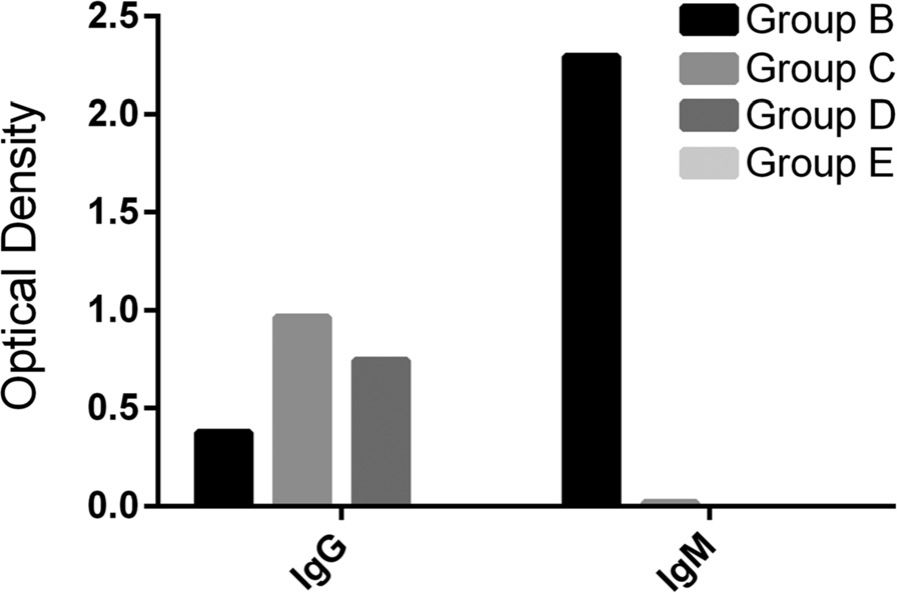
IgG and IgM occurrence in group B, C, D and E

The OD value for IgM in the culture confirmed group B (*n* = 237) was significantly higher (p value, 0.041) than that of control groups C, D and E. There was no significant difference between the control groups D and E (p value, 0.039). Titre value of 3200 for IgM and 200 for IgG, was observed for group B patients (193 of 237) only after ≥3 days of reported fever case, which were undetectable in initial 3 days of feverish condition. Patients of group C (*n* = 237), who were enrolled in medication and recovered, showed significant level of serum IgG (p value, 0.046) but not IgM, when compared to previous data from same patients before medication. For the endemic healthy group D (*n* = 237), the titre for IgG was 400 but with no significant IgM titre value. In case of other disease patients of group E (without *Salmonella* but other infections), the ELISA results were negative for both *Salmonella* anti IgM and IgG (Fig. 2). Serology based typhoid diagnostic tests using sera from the culture confirmed typhoid patients and the control subjects showed that both IgG and IgM based ELISA tests were superior to the Widal TH and TO tests. When sensitivity, specificity, positive predictive value (PPV) and negative predictive value (NPV) of ELISA was compared among group A (*n* = 1371) patients against Widal test, ELISA (IgG/IgM) had higher PPV at ≥1:400 (Table 5). The IgM/IgG titre ≥1:200 had a high sensitivity (95.50 %/96.85 %) and specificity (94.69 %/94.95 %). The diagnostic sensitivity of Widal TO test at titre ≥1/400 was 84.09 % and specificity was 52.65 %. There was a significant difference between case definition for suspected and probable cases of typhoid fever (Table 6).

**Table 5:** Sensitivity, Specificity, PPV and NPV for typhoid fever of ELISA and Widal test in different cut off titers

Test	Titer	Sensitivity	Specificity	PPV	NPV
		95 % CI	95 % CI	95 % CI	95 % CI
IgM	≥1:200	95.50 %	94.69 %	77.66 %	99.09 %
		91.87 % to 97.82 %	93.23 % to 95.92 %	72.24 % to 82.46 %	98.33 % to 99.56 %
	≥1:400	81.23 %	98.29 %	91.77 %	95.70 %
		75.95 % to 85.78 %	97.34 % to 98.97 %	87.45 % to 94.98 %	94.36 % to 96.80 %
	≥1:800	61.97 %	96.48 %	78.38 %	92.50 %
		55.41 % to 68.21 %	95.24 % to 97.48 %	71.74 % to 84.08 %	90.85 % to 93.93 %
	≥1:1600	51.89 %	93.79 %	60.44 %	91.42 %
		44.94 % to 58.78 %	92.24 % to 95.11 %	52.94 % to 67.60 %	89.68 % to 92.95 %
	≥1:3200	47.44 %	92.73 %	54.84 %	90.46 %
		40.61 % to 54.34 %	91.08 % to 94.16 %	47.39 % to 62.13 %	88.65 % to 92.08 %
IgG	≥1:200	96.85 %	94.95 %	78.75 %	99.36 %
		93.61 % to 98.72 %	93.52 % to 96.14 %	73.42 % to 83.45 %	98.69 % to 99.74 %
	≥1:400	85.95 %	98.76 %	93.69 %^**^	97.04 %
		80.92 % to 90.07 %	97.93 % to 99.32 %	89.65 % to 96.51 %	95.89 % to 97.94 %
	≥1:800	62.61 %	97.09 %	81.87 %	92.51 %
		56.12 % to 68.77 %	95.93 % to 97.99 %	75.49 % to 87.18 %	90.87 % to 93.95 %
	≥1:1600	51.89 %	94.48 %	63.22 %	91.48 %
		44.94 % to 58.78 %	93.00 % to 95.72 %	55.59 % to 70.39 %	89.75 % to 93.00 %
	≥1:3200	47.44 %	92.73 %	54.84 %	90.46 %
		40.61 % to 54.34 %	91.08 % to 94.16 %	47.39 % to 62.13 %	88.65 % to 92.08 %
TO	≥1:100	90.32 %	52.41 %	44.22 %	92.12 %
		87.25 % to 92.47 %	49.10 % to 53.91 %	41.18 % to 50.73 %	88.71 % to 94.83 %
	≥1:200	80.95 %	51.77 %	23.29 %	93.76 %
		74.98 % to 86.03 %	48.85 % to 54.68 %	20.27 % to 26.53 %	91.60 % to 95.50 %
	≥1:400	84.09 %	52.65 %	25.34 %	94.54 %
		78.58 % to 88.66 %	49.72 % to 55.57 %	22.22 % to 28.66 %	92.49 % to 96.17 %
TH	≥1:100	80.32 %	53.22 %	48.40 %	90.87 %
		75.28 % to 85.91 %	46.42 % to 55.36 %	42.83 % to 50.27 %	87.14 % to 92.84 %
	≥1:200	72.23 %	58.33 %	38.13 %	89.37 %
		68.24 % to 79.72 %	52.93 % to 64.22 %	31.23 % to 43.10 %	83.11 % to 94.07 %
	≥1:400	62.47 %	55.23 %	39.48 %	90.62 %
		59.23 % to 66.82 %	47.21 % to 59.99 %	31.34 % to 45.83 %	86.92 % to 95.27 %

^**^Highest PPV value

**Table 6:** Sensitivity, specificity, PPV and NPV for typhoid fever of WHO case definition

Suspected case of Typhoid fever	Sensitivity	Specificity	PPV	NPV
	95 % CI	95 % CI	95 % CI	95 % CI
Isolation in Blood culture of;				
*S. typhi*	76.81 %	99.22 %	97.07 %	92.71 %
	71.99 % to 81.16 %	98.47 % to 99.66 %	94.31 % to 98.73 %	91.01 % to 94.18 %
Probable case of Typhoid fever				
TH(1:160)	45.22 %	82.32 %	34.23 %	87.83 %
	41.27 % to 49.17 %	79.91 % to 87.82 %	29.46 % to 38.96 %	81.59 % to 91.31 %
TO(1:80)	43.45 %	82.31 %	34.11 %	87.14 %
	39.29 % to 48.97 %	78.96 % to 84.78 %	31.41 % to 38.85 %	83.72 % to 92.91 %

## Discussion

Typhoid is a major public health problem in third world countries [1,2]. In Nepal, typhoid fever is endemic and the major factors for high prevalence rate include, but are not limited to, illiteracy, poverty, poor sanitation and inadequate facilities for safe drinking water supply. The Widal test based on TO and TH titre values were observed higher in healthy subjects relating to the endemic setting of typhoid fever in Nepal. Even the typhoid relapsed subject group B had significant titre value of TH ≥1:160, describing the persistent anti H in blood sample [11] which also had higher serum IgG but lower IgM antibodies suggesting the late class switching from IgM to IgG in response to the infection well after elimination of pathogen [22]. The sensitivity and specificity of the IgG/IgM ELISA were higher than that of Widal test showing Widal’s incompetence in accurate diagnosis. The cut off titre is more in developing countries compared to developed countries due to sanitary and endemic reasons [23], thus the titre value from Widal test is not accurate enough to picture the complete diagnosis, since the PPV value of TO and TH were too low (0.44 and 0.48 respectively). In developing country like Nepal, the haphazard usage of antibiotic is common thus providing false negative in blood culture, though there were no patients who admitted taking antibiotic within two weeks before enrollment in this study. The false negative Widal test results were probably due to early blood collection before the adequate antibody production [24]. In case of Widal test, which when compared with the WHO case definition, didn’t perform well. The sensitivity dropped by >40 % compared to gold standard blood culture showing that Widal test alone can’t be used as diagnostic approach, though is widely used in rural setting in Nepal till date.

## Conclusion

Higher titer value was observed in healthy endemic population which showed that typhoid diagnosis is biased when Widal test is used alone; however ELISA had more stringent data thus resulting a clear distinction of antibody production against *salmonella*, and making differential diagnosis more accurate among febrile cases. This findings also suggest implementing ELISA on daily routine diagnosis of typhoid fever especially in endemic typhoid area like Nepal.

## Abbreviations

BACTEC: Bactenecin
ELISA: Enzyme Linked Immunosorbent Assay
Peds: Pediatrics
TH: Titer of Salmonella H- Flagellar antigen
TO: Titer of Salmonella H- Somatic antigen
BSA: Bovine Serum Albumin
PBS: Phosphate Buffer Saline
HRP: Horse Reddish Peroxidase
TMB: Trimethyle Benzidine
OD: Optical Density
SD: Standard Deviation
ROC: Receiver Operating Characteristics
CHRB: Capital Hospital Ethical Review Board
PPV: Positive Predictive Value
NPV: Negative Predictive Value
NATRC: Aayurveda Training and Research Center
UGC: University Grant Commission
